# Learning from success cases: ecological analysis of potential pathways to universal access to family planning care in low- and middle-income countries.

**DOI:** 10.12688/gatesopenres.13570.3

**Published:** 2023-01-20

**Authors:** Franciele Hellwig, Aluisio JD Barros

**Affiliations:** 1 International Center for Equity in Health, 1160 Marechal Deodoro St., 3rd floor, Pelotas, RS, 96020220, Brazil; 2 Postgraduation Program in Epidemiology, Federal University of Pelotas, 1160 Marechal Deodoro St., 3rd floor, Pelotas, RS, 96020220, Brazil

**Keywords:** family planning, contraception, reproductive health, health equity, universal access

## Abstract

Background

Universal access to family planning services is a well-recognized human right and several countries and organizations are committed to this goal. Our objective was to identify countries who improved family planning coverage in the last 40 years and investigate which contexts enabled those advances.

Methods

Analyses were based on data from publicly available national health surveys carried out since 1986 in Egypt, Ethiopia, Rwanda, Afghanistan, Brazil, and Ecuador, selected based on previous evidence. We estimated demand for family planning satisfied with modern methods (mDFPS) for each country and explored inequalities in terms of wealth, women’s education, and women’s age. We also explored contextual differences in terms of women’s empowerment, percentage of population living in extreme poverty, and share of each type of contraceptive. To better understand political and sociocultural contexts, country case studies were included, based on literature review.

Results

Patterns of mDFPS increase were distinct in the selected countries. Current level of mDFPS coverage ranged between 94% in Brazil and 38% in Afghanistan. All countries experienced an important reduction in both gender inequality and extreme poverty. According to the share of each type of contraceptive, most countries presented higher use of short-acting reversible methods. Exceptions were Ecuador, where the most used method is sterilization, and Egypt, which presented higher use of long-acting reversible methods. In the first years analyzed, all countries presented huge gaps in coverage according to wealth, women’s education and women’s age. All countries managed to increase coverage over recent years, especially among women from the more disadvantaged groups.

Conclusions

Family planning coverage increased along with reductions in poverty and gender inequality, with substantial increases in coverage among the most disadvantaged in recent years. Policies involving primary health care services, provision of various methods, and high quality training of health providers are crucial to increase coverage.

## Background

Universal access to family planning has been recognized as fundamental to promote gender equality, good health, and well-being ^1– 3^. Family planning can be defined as the capability of women, men, and couples to determine the number and spacing of their children, without any form of discrimination or coercion ^4^. More than to provide knowledge and the means to fertility control, family planning policies help to promote women’s and child’s health ^5^.

Since the 20 ^th^ century, several family planning programs have been launched worldwide, increasing the prevalence of modern contraceptive use and reducing the total fertility rate in several countries ^6^. Among developing regions, higher increases in family planning coverage were found first for Latin America and the Caribbean region, followed by Asia and the Pacific, and Eastern Europe and Central Asia ^7^. In several of these countries, more recent trends are related to reduction of inequalities in coverage, with more vulnerable groups being reached by public policies ^8^. While the more industrialized countries started their fertility transition in the second half of the 20 ^th^ century and rapidly increased their prevalence of contraceptive use, this has been much slower in most African countries. However, some countries have managed to rapidly increase modern contraceptive use, such as Ethiopia and Rwanda, especially since the 2010s, after the 2012 London Family Planning Summit, where commitment with family planning funding and programs was reinforced ^6– 10^.

Several strategies for effective and sustainable family planning policies are already known, such as political commitment, adequate funding, availability of a range of methods, and community leaders ^11^. Based on those, several approaches to address the barriers to increase family planning coverage have been implemented in low- and middle-income countries in the past years, including the promotion of self-administered injection and implants among vulnerable women living in remote areas, and through peer education to reduce contraception stigma among adolescents ^12^. Currently, both lack of knowledge on family planning practices and access to contraceptive methods do not seem to be the main barriers to contraception, even in the world poorest countries ^13^. Instead, family planning is strongly dependent on beliefs and practices based on local social and cultural norms which vary widely across contexts ^13, 14^. Low national coverage and larger gaps have been identified in countries with higher levels of extreme poverty and lower women’s empowerment, where women may face stronger barriers to accessing contraceptives and may be exposed to risky sexual activity ^15, 16^. In addition, in contexts of extreme poverty and limited method mix or untrained health providers, women may prefer sterilization to reversible contraceptives ^17^.

In Africa, especially the West and Central region, the prevalence of contraceptive use is low in most countries. These regions are still strongly affected by social norms of early marriage, desire for large families, and low levels of women’s empowerment ^18^. In addition, most African countries have not provided sufficient resources for family planning in the past decades, resulting in a high level of unmet need for family planning ^19^, especially in Sub-Saharan Africa, where almost 30% of women do not have their need for family planning satisfied ^20, 21^. Coverage of family planning services is even lower among harder-to-reach subgroups, such as young women, women who live in rural areas and who are poor and less educated ^18^. Some countries in Asia also have persistent low levels of family planning coverage ^18^. Low women’s empowerment, social norms, and health system barriers have been recognized as the main obstacles to modern contraception in Asian countries ^22^. Limited knowledge and misconceptions are also important barriers in the region, especially among adolescents ^22, 23^. In Latin America and the Caribbean, high levels of contraceptive use have already been achieved in several countries but remain low in others. In addition, in Brazil, Mexico, Colombia, Dominican Republic, and El Salvador, a large share of demand for family planning is satisfied by permanent methods ^24^, an approach that is increasingly less desirable in terms of sociological aspects now that several long-acting contraceptives are available ^25^. Inequalities in contraceptive use according to socioeconomic and demographic characteristics persist in the region, with the poorer, the less educated, and indigenous women being the most disadvantaged ^2^.

Despite the improvements of the past decades, there is much more to be done. Progress has been much faster in some settings than in others ^7, 8, 26, 27^ and important socioeconomic and demographic inequalities in family planning are still being identified in several low- and middle-income countries ^18, 28^. Our aim was to identify countries who managed to improve family planning coverage since 1980 and investigate which were the contexts that made those advances possible.

## Methods

### Selected geographies

Based on previously published literature ^7, 8, 26, 27^, we sought countries from each of the UNICEF world regions with successful stories of increasing contraceptive use and reducing inequalities. Within each region, we looked up for the countries that presented the largest progress in increasing coverage or the ones that managed to rapidly increase it in the last years. We also considered countries that managed to increase coverage among disadvantaged women, such as the youngest, poorest, least educated, and rural residents. To present a broad picture while limiting the total number of countries in the study, we did not include more than two countries per region. We selected one country from the Middle East and North Africa (Egypt), two from Eastern and Southern Africa (Ethiopia and Rwanda), one from Asia (Afghanistan), and two countries from Latin America and the Caribbean (Brazil and Ecuador).

### Study design and data collection

We used data from publicly available national health surveys, including Demographic and Health Surveys (DHS), Multiple Indicator Cluster Surveys (MICS), and Reproductive and Health Surveys (RHS) carried out since 1986. These surveys are: Afghanistan 2010, 2015; Brazil 1986, 1996, 2006, 2013; Ecuador 1994, 1999, 2004, 2012; Egypt 1995, 2000, 2005, 2008, 2014; Ethiopia 2000, 2005, 2011, 2016, 2019; Rwanda 2000, 2005, 2010, 2014. All surveys included use standardized data collection procedures ^29^.

To increase the amount of information for each selected country we also used estimates provided by the World Bank ( https://data.worldbank.org/) based on other sources of data. To check the consistency of these estimates with the ones based on surveys, we compared existing survey estimates with those published by the World Bank and found no difference in most cases. The comparisons are presented in the supplementary material. A complete list of surveys used in the analyses is presented in Table 1 and underlying data is published at Harvard Dataverse ^29^.

**Table 1.** Demand for family planning satisfied by modern methods in the selected countries and share of modern contraceptive use according to type of method.

Country	Year	Source	mDFPS (%)	short-acting	long-acting	permanent
Afghanistan	2000	WB	16.2	NA	NA	NA
	2003	WB	26.4	NA	NA	NA
	2005	WB	32.1	NA	NA	NA
	2006	WB	38.6	NA	NA	NA
	2008	WB	35.7	NA	NA	NA
	2010	MICS	40.9	84.2	11.7	4.1
	2012	WB	33.9	NA	NA	NA
	2015	DHS	39.4	81.3	8.6	10.2
	2016	WB	41.3	NA	NA	NA
	2018	WB	38.4	NA	NA	NA
Brazil	1986	DHS	79.6	39.7	1.1	59.2
	1996	DHS	89.4	37.6	1.6	60.8
	2006	NSS	91.5	52.1	2.4	45.6
	2013	NSS	93.7	64.7	2.6	32.9
Ecuador	1982	WB	56.0	NA	NA	NA
	1987	WB	52.3	NA	NA	NA
	1989	WB	65.3	NA	NA	NA
	1994	RHS	66.8	30.0	28.1	42.0
	1999	RHS	74.1	37.0	22.3	41.4
	2004	RHS	81.2	41.2	18.6	415
	2012	NSS	89.8	38.8	15.3	45.9
Egypt	1980	WB	52.5	NA	NA	NA
	1984	WB	51.4	NA	NA	NA
	1988	WB	58.8	NA	NA	NA
	1989	WB	58.7	NA	NA	NA
	1991	WB	68.1	NA	NA	NA
	1992	WB	64.0	NA	NA	NA
	1995	DHS	69.3	31.6	65.9	2.5
	1996	WB	66.8	NA	NA	NA
	1997	WB	71.6	NA	NA	NA
	1998	WB	73.2	NA	NA	NA
	2000	DHS	77.3	31.0	66.4	2.6
	2003	WB	78.8	NA	NA	NA
	2005	DHS	79.6	31.8	66.0	2.2
	2008	DHS	80.5	34.8	63.4	1.8
	2014	DHS	80.0	44.0	53.9	2.1
Ethiopia	1990	WB	14.3	NA	NA	NA
	1997	WB	14.0	NA	NA	NA
	2000	DHS	22.2	92.5	2.5	5.0
	2005	DHS	33.8	95.9	2.9	1.2
	2011	DHS	49.7	84.5	13.8	1.7
	2014	WB	55.7	NA	NA	NA
	2016	DHS	60.2	70.5	28.3	1.2
	2019	DHS	63.3	74.0	25.1	0.9
Rwanda	1983	WB	7.0	NA	NA	NA
	1992	WB	32.7	NA	NA	NA
	1996	WB	23.7	NA	NA	NA
	2000	DHS	17.9	76.3	6.0	17.7
	2005	DHS	26.9	89.1	4.9	6.0
	2008	WB	38.8	NA	NA	NA
	2010	DHS	60.8	82.5	15.4	2.0
	2014	DHS	64.3	77.9	18.9	3.1

DHS: Demographic and Health Survey, MICS: Multiple Indicator Cluster Survey, RHS: Reproductive Health Survey, NSS: Non-standard Survey, WB: World Bank data.

### Study population

We evaluated demand for family planning satisfied by modern methods (mDFPS) among women who were married or in a relationship. mDFPS is defined as the proportion of women in need of contraception that were using (or whose partner was using) a modern contraceptive method. Women were considered in need of contraception if they were fecund and did not want to become pregnant within two years or were unsure if or when they wanted to become pregnant. Those who were pregnant at the time of the survey and declared the pregnancy was unintended were also considered in need of contraception. Women were classified as infecund if they were menopausal; had had a hysterectomy; had never menstruated; had had their last period more than six months ago and were not postpartum amenorrhoeic; said they cannot get pregnant; or if they had been married for at least five years, had never used contraception and not become pregnant in the previous five years ^30^.

Different definitions of modern contraceptives have been proposed in the last years ^31, 32^. In this analysis, modern contraceptive methods were defined as medical procedures or technological products ^31^ and included short-acting reversible methods (oral contraceptive pills, injections, spermicides, and male and female condoms); long-acting reversible contraceptives (intrauterine devices (IUD) and implants); and permanent methods (female and male sterilization). This definition does not consider lactational amenorrhea nor any calendar-based method as modern. Although they can be as effective as the methods we are considering as modern, they were not included here since they require a recent pregnancy or couples avoid sex.

### Ethical approval

Ethical clearance was responsibility of the institutions that conducted the surveys, all of them were approved by the national committee of each country. All survey data are anonymized.

### Data analysis

For some surveys, information to identify women in need of contraception was not available. Given the high correlation between demand for family planning satisfied and contraceptive use prevalence, we estimated mDFPS using the following predictive equation ^33^:

$$logit(mDFPS)=0.61+0.68log(CPMO)+3.57CPMO^{2}$$

where mDFPS is demand for family planning satisfied by modern methods and CPMO is the modern contraceptive use prevalence.

CPMO was estimated for all countries without information to estimate mDFPS, considering it as the proportion of women 15-49 years of age currently using a modern contraceptive method. As mDFPS, CPMO was restricted to women who were married or in a union.

The bulk of this study consists of a descriptive analysis of changes in family planning coverage and contextual factors. We used scatter plots to explore changes over time in mDFPS along with changes in the proportion of total population living in extreme poverty (less than US$ 1.90 a day), and in levels of gender inequality. This was measured by the Gender Inequality Index (GII), a composite measure reflecting inequality in achievement between women and men in three dimensions: reproductive health, empowerment, and labor market. A complete description of the index is available elsewhere ^34^. Using survey data, we also evaluated changes over time in mDFPS against changes in the mix of contraceptive methods used in each country and inequalities according to wealth, women’s age, and women’s education. Visual representation of absolute inequalities by each factor was accessed using equiplot graphs (equidade.org/equiplot) while changes in method mix were presented in stacked bar charts.

Wealth was measured based on an asset index obtained from information on household assets, presence of electricity, water supply, sanitary facilities, and building materials of the dwelling, among other variables ^35, 36^. The wealth score was obtained through principal component analyses, estimated separately according to area of residence, since relevant assets may vary in each area, and were later combined into a single score using a regression-based scaling procedure ^35^. The scores assigned to the households were then used to divide them, weighted by the number of residents, into five equally sized groups. Women’s age was categorized in three groups: 15–17 years, 18–19 years, and 20–49 years. Women’s education was classified according to the highest level achieved (none, primary, or secondary/higher).

All analyses were conducted in Stata 16.0 (StataCorp, College Station, TX, USA), always considering the multi-stage survey design, including sampling weights and clustering.

## Results

**Figure 1.**
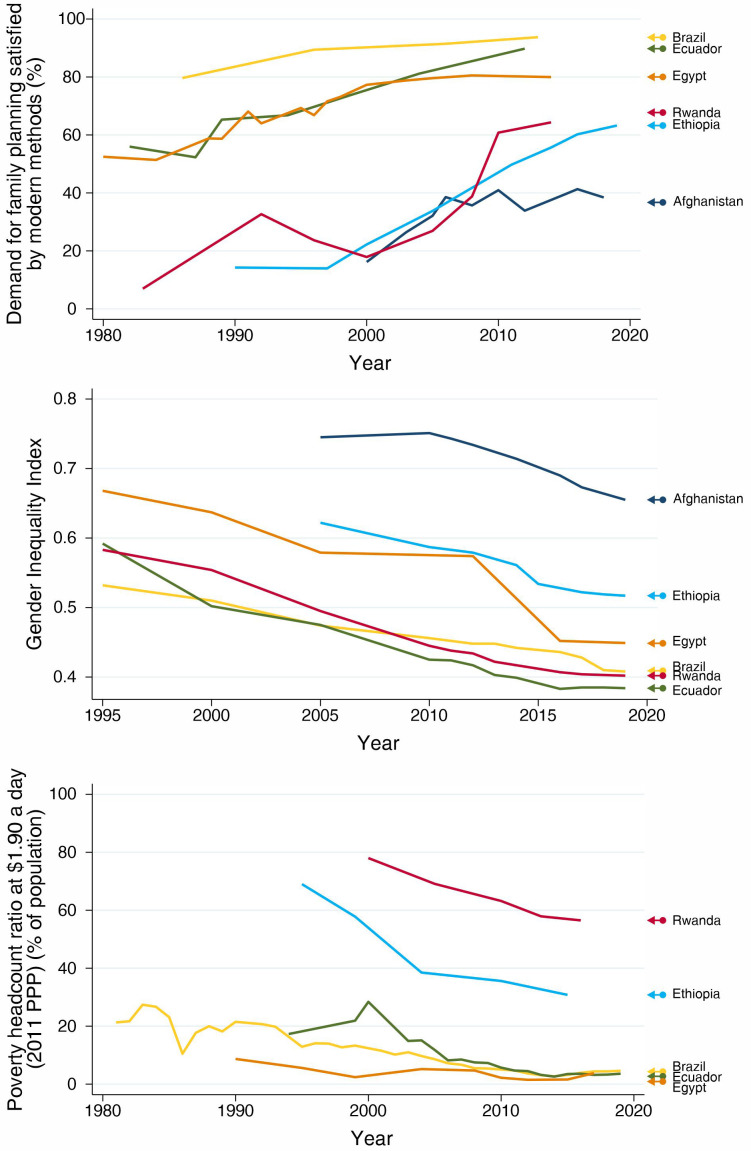
Trends in demand for family planning satisfied by modern methods, Gender Inequality Index, and proportion of total population leaving behind poverty headcount ratio.

In all countries, according to the selection criteria, we observed important increases in mDFPS with time ( Figure 1). The patterns, however, are very distinct. Brazil and Ecuador, from LAC, presented the highest current levels of mDFPS, around 90%. Brazil, Egypt and Afghanistan did not present important changes in the past decade. Brazil has over 90% mDFPS, while Egypt stabilized around 80%. Afghanistan, however, presented a steep increase in mDFPS between 2000 and 2010, but since then coverage stagnated around 40%, with no further progress. Rwanda and Ethiopia presented increasing coverage during the whole study period, finishing with levels slightly over 60%.

Along with the increase in mDFPS coverage, we observed important reductions over time in both gender inequality and the proportion of the population living in extreme poverty in our selected countries. Figure 1 shows that, starting at different levels of gender inequality, all countries had important reductions over time. Interestingly, in Afghanistan the Gender Inequality Index (GII) was stable over the period when mDFPS was increasing and declined after 2010 when mDFPS did not increase any more. Figure 1 presents a similar picture for the proportion of the population living in extreme poverty. Unfortunately, we have no data on the proportion of the population living in extreme poverty in Afghanistan.

**Figure 2.**
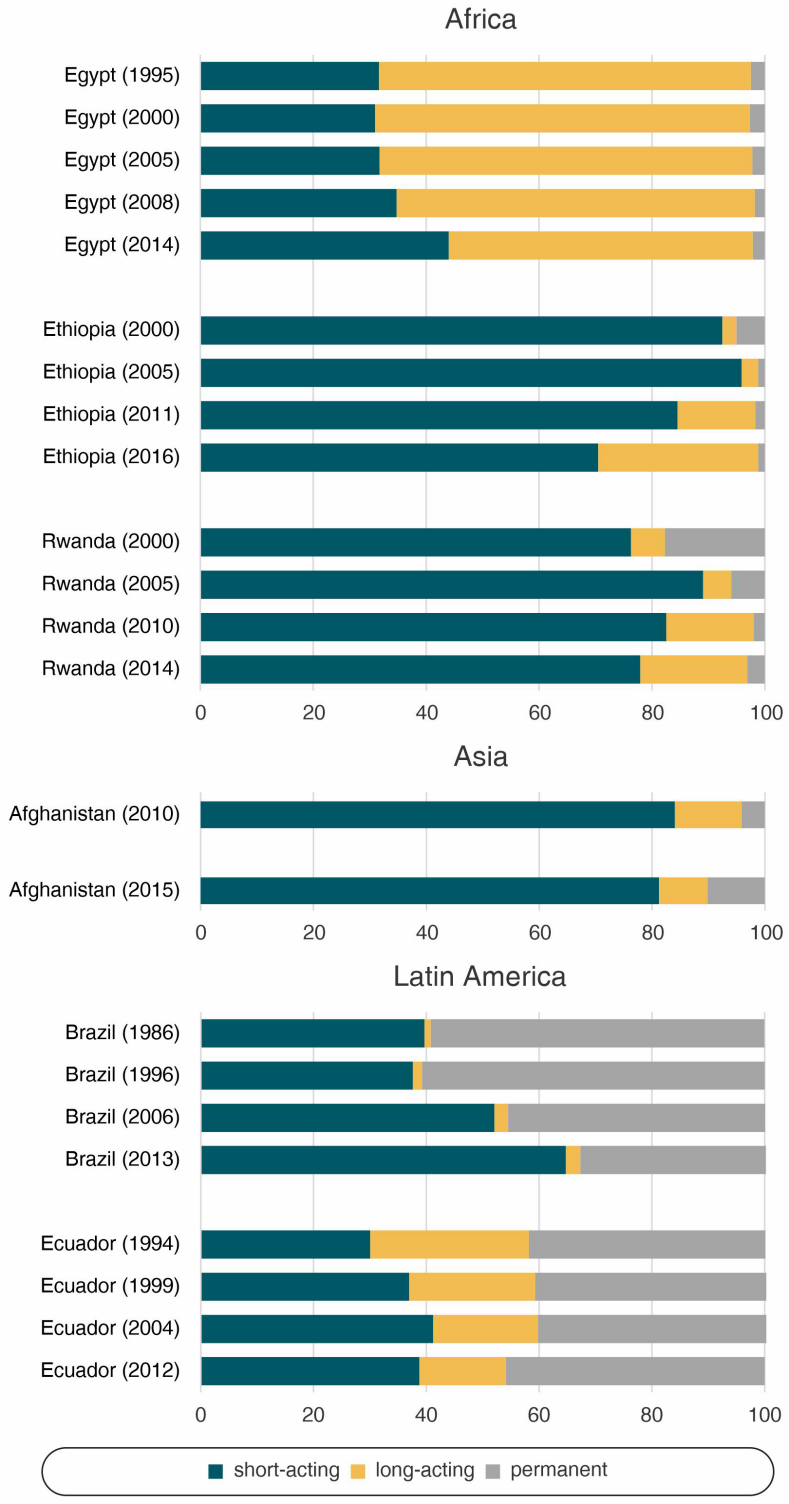
Share of modern contraceptive use according to world region.

The patterns and change in contraceptive method mix were not similar across the study countries ( Figure 2, Table 1). Egypt presented the highest reliance on long-acting contraceptives, while for Ecuador it was permanent contraception. The other countries had a predominance of short-acting methods. Along with Ecuador, Brazil relied heavily on permanent contraception. However, this reliance was reduced over time, a trend that was also observed in Rwanda and to a lesser degree in Ethiopia. The use of long-acting methods increased in Ethiopia and Rwanda, and very discreetly in Brazil. Egypt, Afghanistan, and Ecuador, on the other hand, reduced the share of long-acting reversible methods.

**Figure 3.**
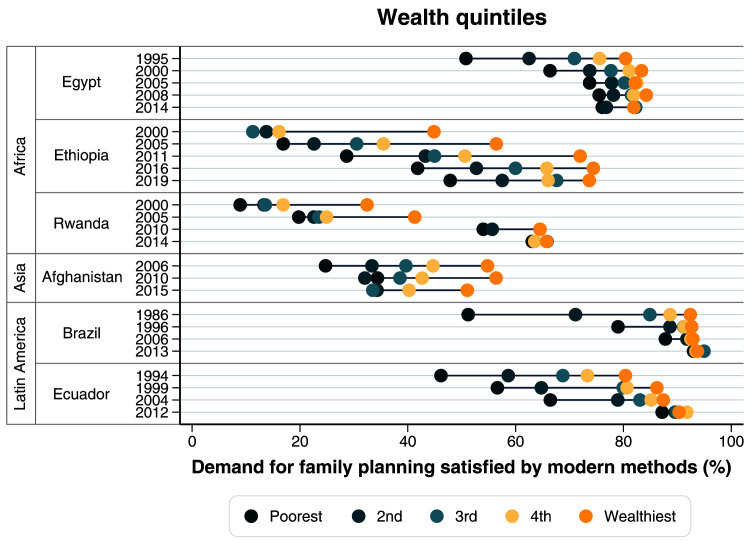
Demand for family planning satisfied by modern methods according to wealth quintiles.

**Figure 4.**
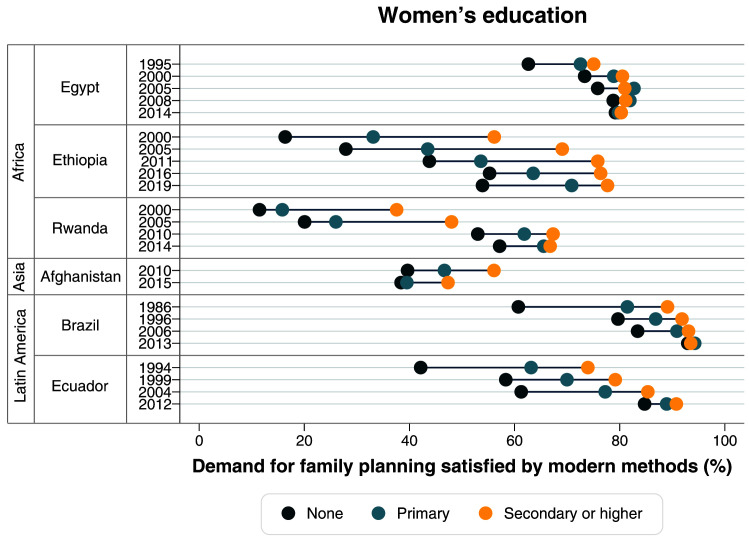
Demand for family planning satisfied by modern methods according to women’s education.

**Figure 5.**
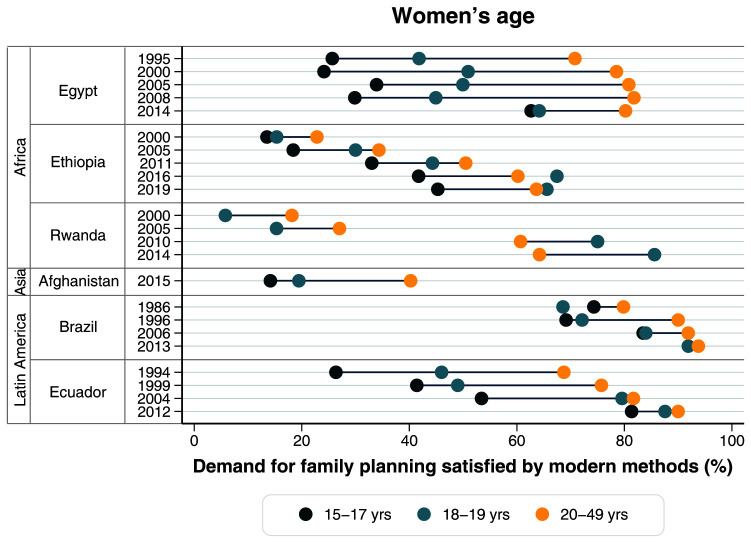
Demand for family planning satisfied by modern methods according to women’s age.

In Egypt, along with a slight reduction in the use of long-acting reversible methods and an increase of short-acting contraceptives between 2008 and 2014 ( Figure 2, Table 1), there was an important reduction in wealth inequalities. Coverage of family planning was already high among the wealthiest in 1995, with a huge gap between the poorest and the wealthiest quintiles. In the last time point, mDFPS was still lower among the poorest than among the wealthiest, but the gap was much reduced ( Figure 3, Table 2). Large gaps in terms of women’s education were also identified in the first surveys, with much lower mDFPS coverage among those with no education. Currently, inequalities in terms of education are virtually null ( Figure 4, Table 3). In terms of women’s age, we observed an important improvement among adolescents, especially in the 15–17 years age group. mDFPS among adolescents is still much lower than among women 20 years or more. However, mDFPS among girls aged 15–17 was less than 30% until 2008 and, in 2014, it presented very important progress, being it 62.7% and nearly matching the coverage for older adolescents (64.2% among girls aged 18–19) ( Figure 5, Table 4).

**Table 2.** Demand for family planning satisfied by modern methods according to wealth quintiles.

Country	Year	mDPFS (%)				
		Poorest	2nd	3rd	4th	Wealthiest
Afghanistan	2006	24.7	33.3	39.6	44.7	54.8
Afghanistan	2010	34.4	32.0	38.5	42.7	56.4
Afghanistan	2015	34.3	34.3	33.5	40.2	51.0
Brazil	1986	51.2	71.1	84.9	88.7	92.4
Brazil	1996	79.0	88.6	91.2	91.3	92.7
Brazil	2006	87.8	91.8	92.7	92.5	92.9
Brazil	2013	93.0	93.2	94.9	93.4	93.7
Ecuador	1994	46.2	58.6	68.8	73.3	80.4
Ecuador	1999	56.6	64.8	79.9	80.7	86.2
Ecuador	2004	66.4	78.9	83.1	85.1	87.4
Ecuador	2012	87.2	89.6	89.8	91.8	90.3
Egypt	1995	50.8	62.5	70.9	75.6	80.4
Egypt	2000	66.4	73.7	77.7	81.1	83.3
Egypt	2005	73.7	77.8	80.2	82.5	82.2
Egypt	2008	75.5	78.1	81.5	81.9	84.2
Egypt	2014	76.1	76.8	82.3	81.9	81.9
Ethiopia	2000	13.8	13.8	11.3	16.1	44.9
Ethiopia	2005	16.9	22.6	30.5	35.5	56.4
Ethiopia	2011	28.7	43.3	45.0	50.6	72.0
Ethiopia	2016	41.8	52.7	60.0	65.8	74.4
Ethiopia	2019	47.9	57.5	67.6	66.0	73.7
Rwanda	2000	8.9	13.3	13.6	16.9	32.4
Rwanda	2005	19.8	22.6	23.5	24.9	41.3
Rwanda	2010	54.0	55.7	64.5	64.5	64.5
Rwanda	2014	63.1	63.4	65.8	63.5	65.7

**Table 3.** Demand for family planning satisfied by modern methods according to women’s education.

Country	Year	mDFPS (%)		
		None	Primary	Secondary +
Afghanistan	2010	39.6	46.7	56.1
Afghanistan	2015	38.4	39.5	47.3
Brazil	1986	60.7	81.5	89.1
Brazil	1996	79.7	86.8	91.9
Brazil	2006	83.4	90.9	93.1
Brazil	2013	93.0	94.3	93.5
Ecuador	1994	42.1	63.2	74.0
Ecuador	1999	58.3	70.0	79.2
Ecuador	2004	61.3	77.2	85.4
Ecuador	2012	84.7	88.9	90.8
Egypt	1995	62.6	72.5	75.1
Egypt	2000	73.3	78.9	80.5
Egypt	2005	75.8	82.7	81.0
Egypt	2008	78.8	81.9	81.1
Egypt	2014	79.2	79.8	80.3
Ethiopia	2000	16.4	33.1	56.1
Ethiopia	2005	27.9	43.5	69.1
Ethiopia	2011	43.8	53.6	75.8
Ethiopia	2016	55.3	63.5	76.4
Ethiopia	2019	53.9	70.9	77.7
Rwanda	2000	11.5	15.8	37.6
Rwanda	2005	20.1	26.0	48.0
Rwanda	2010	53.0	61.9	67.3
Rwanda	2014	57.2	65.6	66.8

**Table 4.** Demand for family planning satisfied by modern methods according to women’s age.

Country	Year	mDFPS (%)		
		15–17 yrs	18–19 yrs	20–49 yrs
Afghanistan	2015	14.2	19.5	40.3
Brazil	1986	74.3	68.6	79.8
Brazil	1996	69.2	72.1	90.0
Brazil	2006	83.5	84.0	91.9
Brazil	2013		91.9	93.8
Ecuador	1994	26.4	46.0	68.8
Ecuador	1999	41.4	49.0	75.7
Ecuador	2004	53.4	79.6	81.7
Ecuador	2012	81.3	87.6	90.0
Egypt	1995	25.7	41.8	70.8
Egypt	2000	24.1	50.9	78.5
Egypt	2005	33.9	50.0	80.8
Egypt	2008	29.9	44.9	81.8
Egypt	2014	62.7	64.2	80.2
Ethiopia	2000	13.5	15.3	22.8
Ethiopia	2005	18.4	30.0	34.4
Ethiopia	2011	33.0	44.3	50.5
Ethiopia	2016	41.7	67.5	60.2
Ethiopia	2019	45.3	65.6	63.6
Rwanda	2000		5.8	18.2
Rwanda	2005		15.3	27.0
Rwanda	2010		75.0	60.7
Rwanda	2014		85.6	64.2

Between 2000 and 2016, mDFPS in Ethiopia increased from 14.3% to 63.3% ( Figure 1, Table 1), with an increase in the use of long-acting reversible contraceptives and a reduction in permanent contraception ( Figure 2, Table 1). Examining how wealth inequalities changed over time, we observed reduction in the gap between the poorest and the richest, but a large gap still persists ( Figure 3, Table 2). Among the poorest, mDFPS coverage increased from 13.8% in 2000 to 47.9% in 2019. Over time we also observed an important change in the patterns of inequality ^37^ – from a very clear top inequality situation in 2000, to a linear pattern in 2019. A large reduction in the gap between levels of education was also observed ( Figure 4, Table 3). In terms of age, the gap actually increased, with the youngest women now significantly trailing behind the others ( Figure 5, Table 4).

Rwanda made impressive progress in mDFPS, despite some decrease in coverage in the 1990s, which may be partly due to the use of different data sources in our analysis. From 2000 to 2014, mDFPS increased 3.6 times, from 17.9% to 64.3% ( Figure 1, Table 1). The increase in coverage was accompanied by an increase in long-acting reversible contraceptives and a decrease in sterilization ( Figure 2, Table 1). The change in wealth inequalities was most impressive, with a large gap and a top inequality pattern in 2000 being replaced by essentially no wealth inequality in 2014 ( Figure 3, Table 2). In terms of women’s education, the gap also reduced, with increased mDFPS coverage in all groups. However, mDFPS is still lower among those with no education, with 57.2% mDFPS, while women with secondary or higher education are at 66.8% ( Figure 4, Table 3). In terms of age, the gap actually increased, but remarkably mDFPS started higher for women 20+ years, but from 2010 this pattern flipped and in 2014 adolescents 18–19 years presented a much higher coverage of 85.6% ( Figure 5, Table 4).

Despite its weak track record in gender equality, Afghanistan succeeded in increasing family planning for a period. Even before its commitment with the Family Planning 2020 initiative in 2016, its mDFPS coverage increased from 16.2% to 38.4% between 2000 and 2018, but most progress was achieved between 2000 and 2005 ( Figure 1, Table 1). With only two available time points to assess the method mix, it is clear that short-acting reversible methods are by far the most used. There was some increase in permanent methods (4.1% to 10.2%), while long-acting contraceptives decreased ( Figure 2, Table 1). Large inequalities in mDFPS coverage still exist in terms of wealth, education, and age ( Figure 3– Figure 5). Most notably, younger women are far behind in mDFPS compared to those 20 years and over.

In 1986, Brazil already had a high mDFPS coverage of 79.6%, with permanent contraception being the most common type. Large inequalities were present then, according to wealth, women’s age and women’s education ( Figure 3– Figure 5). Over time, impressive progress was achieved, with inequalities in all these dimensions decreasing to virtually null, while overall mDFPS reached 93.7% in 2013. The share of sterilization decreased and was just over 30% in the last time point.

In Ecuador, mDFPS coverage increased from 56.0% % in 1982 to 89.8% in 2012 ( Figure 1, Table 1). Permanent contraception are currently the most used methods with 45.9% of the share, having increased in the last period ( Figure 2, Table 1). Ecuador had huge and persistent inequalities in mDFPS in terms of wealth, education, and women’s age up to 2004 ( Figure 3– Figure 5). In 2012 these inequalities nearly disappeared for all dimensions with younger adolescents presenting 81.3% of mDFPS coverage, up from 53.4% in 2004 ( Figure 5, Table 4).

## Discussion

The objective of this paper was to identify characteristics, actions and programs in successful countries that may contribute to increasing family planning coverage in other settings. Our findings suggest a concomitant improvement in mDFPS coverage, gender equality, and reduction in poverty, with the more recent data indicating faster increases in coverage among the more disadvantaged women.

In agreement with our findings, several previous studies have identified a positive association between women’s empowerment and family planning coverage ^38– 40^. Gender equality is a sexual and reproductive health determinant at the individual, family, and social levels, influencing women’s decision-making power, mobility, financial autonomy, spousal communication, freedom from control by partner or family, exposition to intimate-partner violence, their aspirations, level of education, and their participation in the labor market ^38– 40^. At the same time, family planning has also been recognized as a relevant strategy to empower women and engage them in economic activities ^41^. A cyclic relationship is also experienced in terms of economic development, in which ensuring access to family planning services has been recognized as a relevant strategy to alleviating poverty, Lower levels of family planning coverage are persistently identified among poorer women, with lower availability of contraceptive methods, less trained providers, and higher perceived costs often identified in lower-income settings ^41, 42^. The reduction in the proportion of the population living in extreme poverty that we identified along with the increases in the levels of mDFPS, illustrates this long-term effect of the improvement in living conditions and increase access to family planning services.

To evaluate pathways to success in family planning, we faced several limitations regarding the availability of information, specially related to family planning funding. It is known that several countries and international organizations have made financial commitments to increase family planning coverage, especially since the early 2010s ^6^. Unfortunately, information on health expenditure specifically on reproductive health is not available for most of the countries included in our analysis ^43^. Another limitation is related to women’s empowerment. Although a measure of women’s empowerment using national health surveys was already developed ^44, 45^, all the required information to estimate it is not available in most of the surveys included in our study. For this reason, we chose to use the Gender Inequality Index as an indicator of empowerment. Our study was also limited because information for longer periods is not available for all selected geographies and there are other countries that managed to increase family planning coverage but there is no available data to explore the context of these changes, especially those in Asia. Some of the aspects related to successes in family planning coverage which we could not measure in our article were already discussed in previous studies. Major policies and contributors in each setting are presented below, according to each country case.

### Country cases

***Egypt.*** Egypt, an Arab country with historical cultural norms regarding early marriage and large families, began its commitment with family planning practices aiming to control population growth due to the narrative of its negative effects on availability of resources and national development ^46^. Egypt started to limit its population growth in the 1930s and in the 1950s the government started its endorsement of birth control and modern contraceptives were increasingly available ^46, 47^. With a high level of coverage since the 1980s, part of the Egyptian success in increasing contraceptive use was due to an early agreement between Western donors, national health professionals and female activists who managed to increase public trust and women’s demand for family planning ^46^. The involvement of different leaders led to the promotion of family planning in community contexts and health facilities, integrating family planning with both health and social services ^46^. Another differential of Egyptian family planning policies was that family planning messages were not designed in favor of couples’ choices regarding family size, but were in favor of smaller families ^48^.

Egypt decreased its total fertility rate of 5.6 births per women in 1976 to 2.8 in 2007 ^46, 49^. Between the 1970s and the early 2000s, in a context of political instability, the number of health facilities have increased over 50 percent and the resources allocated by national government to family planning services have increased by 400 percent, contributing to an increase in the contraceptive use prevalence from 19% in 1976 to 59% in 2005 ^49, 50^. The reduction in fertility rate was largely credited to the increased use of contraception, and in a smaller measure to the increase in the number of induced abortions and the increase in the age of marriage ^48, 49^.

In the family context, factors identified as important determinants of contraceptive use were the desire for less children, the number of living children, place of residence, woman’s work after marriage, and the level of education of the woman and her husband ^50^. Although we found a high level of gender inequality until the 2010s, previous studies indicated that Egyptian families have been built upon more equitable standards. In 1992, men and women already had similar fertility preferences, with an ideal family size of three children on average ^50^. While in 1992, only 29% of Egyptians declared that there was an agreement in fertility preferences ^50^, in 2008, already more than 85% of women using modern contraceptives had declared that this decision was made jointly with their husbands ^48^. Despite the progress in women’s education, female employment and wife’s opportunity cost did not lead to a significant lower number of wanted children during the peak of increase in modern contraception ^48^. More recent studies have found a significant effect among women with secondary level or higher ^51^, however, the early adoption of family planning policies seems to be a stronger factor in the desire for smaller families and modern contraceptive use among women of all socioeconomic groups in Egypt ^48^.

***Ethiopia.*** Ethiopia is one of the most populous countries in Africa, which had been exposed to huge political instability in the second half of the 20 ^th^ century, with the abolishment of the parliament, domain of an authoritarian revolutionary regime, suspension of the constitution, and land expropriation ^52, 53^. Since the 1980s, Ethiopians have been facing water scarcity and repeated famine episodes. The critical scenario naturally affected the desired family size in Ethiopia. Following this increased demand for contraceptives and in partnership with international donors, Ethiopia managed to increase provision of contraceptive methods and, consequently, the national coverage raised. Modern contraceptive use prevalence increased from 2.9% in 1990 to 27.3% in 2011, and total fertility rate declined from 7 children per woman to 4.8, respectively ^54^.

The first movement related to family planning policies in Ethiopia occurred in 1966, with the foundation of the Family Guidance Association of Ethiopia, affiliated with the International Planned Parenthood Federation. The first national policy was implemented in the early 1990s and, as in Egypt, its primary concern was to reduce the population growth to promote socioeconomic development. Aspects addressed in this policy were the elimination of legal barriers to socioeconomic rights for women and family planning propaganda advising in favor of smaller families. The following policies expanded the sources of contraceptives and proposed new plans to end poverty and expand the number of health providers and sources of contraceptives ^54, 55^. Structural factors such as the number of modern contraceptive methods available and distance to health facilities have been identified as significant factors associated with increased use of contraception among Ethiopian women ^56^. Family planning was also included in HIV, postabortion and postpartum services ^54, 57^. Later on, in the early 2000s, the national government launched the Health Extension Plan, which delivers primary health care and family planning services in the more vulnerable settings, and has removed import taxes to contraceptive methods ^54, 57^. Since 2009, health extension workers were allowed to provide implants and midwives to insert IUDs ^58, 59^. It was a successful strategy, which was fundamental for the increase of long-acting reversible methods. In the 2012 London Family Planning Summit, Ethiopia put family planning in the core of its health system, aiming to address aspects related to supply of contraceptives, increase of the family planning budget, reduce early marriage, and improve its strategy to meet the needs of adolescents ^54, 60^. Complementary to the health extension workers, school-based family planning programs have being providing sexual and reproductive education to girls and reinforcing the importance to use contraception and continue education ^60^.

Among the selected countries, Ethiopia was the leading country in reductions in the proportion of the population living in extreme poverty and managed to increase mDFPS among women from all groups of age, wealth, and education. The progress made in Ethiopia was also made possible by the international donor support and by the support provided by nongovernmental organizations, improving service delivery and promoting behavior-change campaigns. Because of its delicate situation and the national government commitment with family planning, Ethiopia was the African country that received most international funding for family planning. In addition to monetary resources, Ethiopia has been receiving technical and management resources from the Global Health Initiative ^54^.

***Rwanda.*** Between 2005 and 2015, Rwanda increased its modern contraceptive use from 17% to 53% ^61^ and decreased its total fertility rate from 6.1 to 4.6 births per woman ^62^. The major factor that possibly contributed its success was the government commitment, who increased the family planning budget and made family planning services available ^54, 61– 63^. Family planning services in Rwanda are still being funded mostly by international organizations ^61^, but the national government made them a national priority and, with collaboration of different sectors, innovation and evidence-based strategies, have been implementing and supporting family planning policies ^63, 64^.

The discussion on promotion of contraception started in Rwanda much later than in Egypt and Ethiopia, in the early 1980s, with the creation of the National Office of Population ^64^. National family planning policies in Rwanda have been built upon strong campaigns with training of providers, increase of the range of methods available, and mass media campaigns ^62^. Aiming to improve reproductive health outcomes and endeavor national development, the creation of the National Reproductive Health Policy in 2003 addressed issues related to women’s, adolescent’s and child’s health, prevention of sexually transmitted infections, family planning, and women’s decision-making power ^63, 65^. With the 2005 National Policy for Family Planning and the 5-year strategy, other important aspects were addressed in order to increase family planning coverage, such as the encouragement of the participation of men and the whole community in family planning discussions, increased efficiency in the provision of family planning services, construction of more health posts, facilitated distribution of short-acting reversible methods by community health workers, and the promotion of training to insertion of long-acting reversible methods and male permanent contraception ^61, 62^. These policies are aligned with our findings that between 2005 and 2010 Rwanda achieved not only important increases in mDFPS, but also reductions in gaps in coverage and a more balanced mix of methods, with a reduction in the role of sterilization and an increase in the share of long-acting methods. Another potential contributor for this improvement was the decentralization of health services, with increased access to health services for those living in rural areas ^61, 63^.

The increased number of women in the parliament has suggested that part of Rwanda’s success in family planning programs is related to gender-equality issues at a macro level ^65^. However, since the start, the main aim of family planning policies in Rwanda has been to reduce population growth, not to tackle gender equality ^62, 63, 65^. Pursuing the government aim to transform Rwanda into a middle-income country by 2020, family planning messages have been putting smaller families not only as contributive but as imperative to reduce poverty and promote development ^62, 63, 65^. On the other hand, along with the provision of family planning services, education, job opportunities, and empowerment of women were promoted by national policies in order to support behavior changes regarding fertility preferences ^64^.

Previous studies indicate that individual factors associated with greater use of contraception among Rwandese women were their level of education, place of residence, agreement with their husband regarding the desired number of children, experience of child mortality, and exposure to family planning information ^62^.

***Afghanistan.*** The impressive progress observed for the other selected countries was not observed for Afghanistan. Even so, it was the country with the fastest increase among the Asian countries with available data. The Islamic Republic of Afghanistan is a country with strong religious and strict social norms, and it has been ravaged by war and plagued by political instability under the Taliban from 1996 to 2001 ^66, 67^. Despite the issues in its health and education system, the country managed to increase family planning coverage in the past two decades, after the deposition of the Taliban regime, in 2001, with the US led invasion of the country ^66, 67^ and the implementation of important strategies. Important factors associated with this success were the engagement of different members of the community in family planning discussions, the focus on the benefits of birth spacing to the health of children and mothers, literacy programs for women, and the increase in the number of female community health workers ^68^.

In the late 1990s, Afghanistan had a total fertility of 7.5 children per woman and one of the highest rates of maternal mortality in the world ^67^. With the family planning messages focusing on the importance of larger birth spacing, parity started to decline and the age of first childbearing started to increase in the early 2000s ^66^. The first national health policy was implemented in 2003, the Basic Package of Health Services, which aimed to deliver a variety of health services, including family planning ^66^. The higher acceptance of family planning among Afghan families is probably due to its specific approach, that was more sensitive to the health benefits of larger birth spacing than to the potential economic benefits of smaller families ^68^.

Differences in acceptance of contraception between different ethnic groups in Afghanistan has been documented ^67^. Despite the huge heterogeneity, increase in contraceptive use has been documented in regions where religious leaders supported it ^68, 69^. In some settings, they were also providing family planning knowledge to men. This represents a very important advance, since Islam is not only the predominant religion, but the foundation of their culture and their lives ^68^. Despite the religious concerns regarding family planning, Islam allows it when pursuing the common good or when the family is very poor ^68, 69^.

Strong cultural gender inequalities are another barrier to contraceptive use, due to the preference of a male child, which tends to increase the number of children, and due to power imbalance between husband and wife ^68, 69^. Another barrier to contraception is the influence of other family members in the desired family size. Life experiences of older family members tend to be passed on to current generations ^69^.

Important strategies to deal with these barriers were the promotion of basic education to women, the support from non-government organizations, family planning services working with both men and women, the integration of family planning with other health services, and the implementation of community health workers ^68^.

Despite the improvement in family planning coverage starting in 2000 with the end of the Taliban rule, there was much space to increase coverage, which stalled after 2008. Several basic aspects were not addressed, such as the lack of male involvement in family planning counseling, limited method mix offered in public facilities, limitation of health providers to offer specific methods, such as IUD and injectables, and religious prohibition of some contraceptive methods ^69^. Unfortunately, the progress that was achieved in the previous decades is now being lost after the Taliban regained power in 2021 ^70^. Notwithstanding, we opted to keep Afghanistan in our study since our analysis was in an advanced stage in 2021. It is important to note, however, that the country can no longer be considered a success story.

***Brazil.*** Public policies related to population growth started in Brazil in the 1950s, in a context of high fertility rate and fears of a demographic explosion ^71, 72^. Only at the end of the 20th century was the impact of family planning on women’s health included in the official discourse ^72^. Contrary to the other countries selected and despite the international pressure for population control, the Brazilian government was not directly involved in the first family planning programs ^73, 74^. The first reproductive health policy from the government, the Program of Integrated Assistance to Women’s Health, was only launched in 1986 ^74, 75^.

During the 1960s, in addition to the rapid population growth and in a time when contraception was considered a taboo in the country, Brazil had a high rate of induced abortions. In a context where promotion of contraception was out of the law, the high occurrence of abortion was a powerful motivator to private health providers to offer contraceptives ^71, 74^. Despite the prohibition of contraception according to the 1941 Act, condoms were allowed to prevent diseases and contraceptive pills were allowed for ovulation control and regularization of the menstrual cycle ^71^. In a context of industrialization and increasing female insertion in the labor market, the *Sociedade Civil Bem-estar Familiar no Brasil* (BEMFAM, Society for Family Wellfare) was founded in 1965, with support from the International Planned Parenthood Federation, aiming to open the discussion on reproductive health, increase the provision of family planning services, and provide training to health professionals ^71^. The press, through news and analysis articles on family planning, the TV generally, and especially through the popular soap operas showing wealthier families with small families may also had a role in behavior change and promotion of family planning in Brazil ^71, 76^.

During the second half of the 20 ^th^ century, socioeconomic conditions had improved, social mobility had increased, consumption expectations had raised, and national public health started to migrate from control of diseases to hospital-based curative care, leading to a growing demand for female sterilization, which was mostly performed after a cesarian section and paid out-of-pocket. In the more vulnerable regions, sterilizations were paid by politicians, in exchange for votes ^73, 77^. Between 1978 and 1986, use of sterilization increased more than 100 percent in the Southeast region and almost 80 percent in the Northeast region ^74^. In 1986, more than half of the married women were already using a modern contraceptive method, mostly female sterilization or the contraceptive pill ^74^. Use of sterilization continued to increase over the 1990s, when it became subsidized by the Brazilian Unified Health System created by the new 1988 constitution ^74^. Up to 2013, Brazil managed to increase family planning coverage to 94% and reached all population subgroups. As our initial analysis indicated, there was a reduction in the share of permanent contraception in favor of short-acting methods, but long-acting contraceptives are still little used in the country ^75, 78^.

***Ecuador.*** Aiming to improve maternal and child health and guarantee families’ rights to plan their family size, family planning was made one the highest priorities in Ecuador since the 1970s. Between 1970 and 2015, modern contraceptive prevalence increased from 15% to 61% ^79^. The national commitment with the human right of families to choose their family size and space births is demonstrated in the 1998 Ecuadorian constitution and in the National Population Policy of 1987 ^80^. The USAID played a major role in the initial years of the Ecuadorian reproductive health programs, between 1970 and 1999, working with public and private institutions and getting support from other international organizations. The aim of the policies was to increase use of family planning services and improve maternal and child health with sustainability, aiming to increase the financial sustainability and independence of major local nongovernmental organizations, *the Associación Pro-bienestar de la Familia and the Centro Médico de Orientación y Planificación Familiar* ^80^.

Despite the efforts made in the first decades of family planning policies in Ecuador, more disadvantaged women were not reached. Although mDFPS was already high among the more advantaged groups in 1994, until 2004 coverage was low among the poorest, less educated, and younger women. The scenario changed with the health reform in 2007, when the supply of modern contraceptive methods increased in primary health care facilities and the offer of female sterilization increased, especially after childbirth ^81^. Trying to reach adolescents, health services for adolescents were first differentiated, with health providers being trained to be more sensitive to adolescents needs and language. Because it was not resolute, in a second stage, the Intersectoral Policy for the Prevention of Pregnancy in Girls and Adolescents was created, aiming to address social and contextual barriers to contraceptive use ^82^.

Despite the increase in coverage among those who were poorer and who lived in more remote areas ^83^, there is evidence of the insufficient effect of this increase in the supply of contraceptives on modern contraceptive use, especially among indigenous women, due to lack of cultural sensitivity ^81, 84^. Other persistent barriers to increase coverage among more disadvantaged populations are the gender-based violence and absence of economic opportunity ^81^.

## Conclusions

Over the 20 ^th^ century several countries managed to increase modern contraceptive use and decrease their fertility rates based on the ideas of avoiding a demographic explosion and promoting national development and economic growth. In the 21 ^th^ century, there was wide perception that the ideal number of children for a family is not a decision for international or governmental organizations to make. Family planning involving the number and timing of children should be decided by the woman and the couple, according to their needs and desires. Society is not a unique agent with a unique aspiration, but an aggregate of different individuals and different aspirations.

Despite the improvements made in the selected countries, in most of them there is space for more improvement, especially among the more disadvantaged groups. Aspects highlighted are the natural expansion of coverage with the expansion in the proportion of the population living in urban areas, and the better integration of family planning services in other health services ^54, 56^.

Obviously, we cannot replicate the same strategies to different cultural and socioeconomic contexts. However, some basic aspects were fundamental to increase coverage in the geographies analyzed and could be beneficial to other settings. Crucial factors to increase coverage were governmental commitment with well-designed policies and the involvement of primary health services. It is also essential that trained health providers are equipped to offer precise and clear information on family planning and on all the available methods. Also, a wide mix of methods must be available to match the needs and preferences of both women and men ^63^. Policies and approaches should also be culturally adapted to offer acceptable alternatives to different groups, be them religious or ethnic. Finally, a strong commitment of all society stakeholders must be made in order to make family planning available to all, so that no one is left behind ^61^.

## Data Availability

Data used in this study are from: The women’s dataset of Afghanistan 2015; Brazil 1986, 1996; Egypt 1995, 2000, 2005, 2008, 2014, Ethiopia 2000, 2005, 2011, 2016, 2019; and Rwanda 2000, 2005, 2010, 2014, available from the Demographic and Health Survey (DHS) website. Access to the dataset requires registration and is granted only for legitimate research purposes. A guide for how to apply for dataset access is available at: https://dhsprogram.com/data/Access-Instructions.cfm The women’s dataset of the Afghanistan 2010 Multiple Cluster Indicator Survey (MICS), available from the MICS website. Access to the dataset requires registration and is granted only for legitimate research purposes. Questions about data access can be directed to mics@unicef.org The women’s dataset of Ecuador 1994, 1999, 2004 Reproductive Health Survey (RHS), available from the CDC website. Access to the dataset requires registration and is granted only for legitimate research purposes. The women’s dataset of Brazil 2006, 2013, available from the *Pesquisa Nacional de Saúde* website. Access to the dataset requires registration and is granted only for legitimate research purposes. The women’s dataset of Ecuador 2012, available from the *Encuesta Nacional de Salud y Nutrición* website. Access to the dataset requires registration and is granted only for legitimate research purposes. Data on the family planning coverage were obtained from the World Bank for Afghanistan 2000, 2003, 2005, 2006, 2008, 2012, 2016, 2018; Ecuador 1982, 1987, 1989; Egypt 1980, 1984, 1988, 1989, 1991, 1992, 1996, 1997, 1998, 2003; Ethiopia 1990, 1997, 2014, 2016, and Rwanda 1983, 1992, 2008. World Bank data is available for open access. Demand for family planning satisfied by modern methods coverage is available at: https://data.worldbank.org/indicator/SH.FPL.SATM.ZS Harvard Dataverse: Demand for family planning satisfied in successful countries. https://doi.org/10.7910/DVN/HKZLOS ^29^.  This project contains the following underlying data: raw data.csv (all estimates generated from the above listed sources) This project contains the following extended data: raw data - supp material.tab (Comparison between International Center for Equity in Health estimates for modern contraceptive use and data from World Bank) Data are available under the terms of the Creative Commons Zero "No rights reserved" data waiver (CC0 1.0 Public domain dedication). Demand for family planning satisfied by modern methods in selected countries Data from publicly available health surveys, standardized by the International Center for Equity in Health/Pelotas, Brazil
