# Supplementary material for: Learning from success cases: ecological analysis of potential pathways to universal access to family planning care in low- and middle-income countries
Source: Gates Open Res. 2023 Jan 20;6:59. Originally published 2022 Apr 29. [Version 3] doi: 10.12688/gatesopenres.13570.3 (PMC9873636; doi:10.12688/gatesopenres.13570.3)
Supplement: Peer review history

# Peer review: Reviewer response for version 3

**DOI:** 10.21956/gatesopenres.15416.r32855

**Published:** 2023-01-24

**Authors:** Lynne Gaffikin

**Affiliations:** 1 School of Medicine, Stanford University, Stanford, CA, USA

After reviewing this latest version with its various changes, my recommendations have been successfully/adequately addressed and my final assessment is approved for indexing. The only comment I would make is that there are a few typos/syntactical errors remaining that need to be corrected before final online posting.

Is the work clearly and accurately presented and does it cite the current literature?

Partly

If applicable, is the statistical analysis and its interpretation appropriate?

Yes

Are all the source data underlying the results available to ensure full reproducibility?

Yes

Is the study design appropriate and is the work technically sound?

Partly

Are the conclusions drawn adequately supported by the results?

Partly

Are sufficient details of methods and analysis provided to allow replication by others?

Yes

Reviewer Expertise:

Public health practitioner and epidemiologist with expertise in factors affecting family planning programming, barriers and use in low-resource settings globally.

I confirm that I have read this submission and believe that I have an appropriate level of expertise to confirm that it is of an acceptable scientific standard.

# Peer review: Reviewer response for version 2

**DOI:** 10.21956/gatesopenres.15400.r32687

**Published:** 2022-10-18

**Authors:** Lynne Gaffikin

**Affiliations:** 1 School of Medicine, Stanford University, Stanford, CA, USA

**Question 1: Is the work clearly and accurately presented and does it cite the current literature?**

 The work seems accurately presented given the data sources, analysis findings and references cited. However, I would consider modifying the title to say *potential* pathways. 

 The keywords listed are family planning, contraception, reproductive health, health equity and universal access. While family planning is a known contributor to reproductive health, I would consider excluding reproductive health as a keyword as the article focuses on family planning/contraception. Also, the article focuses considerably on gender and contraceptive use inequality which I would suggest as more appropriate under keywords than health equity.

 Regarding the references, #31 and #33 are the same – is this intended? And, reference #67 needs correcting. It says Bertrand BJT; it should be read as Bertrand JT.

 In terms of clarity, the article notes that the reason for selecting the 6 countries was based on previous evidence including reference to a 2018 sister publication (on which the two authors were also co-authors) that looked at countries lagging behind on contraceptive use (Ewerling *et al.*, 2018 ^1^).

 Of note, Table 1 from reference #8 does not include Ethiopia or Rwanda - two countries in the current analysis - as having the highest mDFPS of countries in that region. There are good reasons why the 6 countries were selected but as 6 is a small number, and the paper points to these countries as success stories, it would be useful to have more details on the rationale for their selection of these 6.

 Throughout the article the authors refer to vulnerable groups, disadvantaged groups and vulnerable areas. Vulnerable is thus one term that is not clearly defined early in the article nor consistently used. The sister article notes subgroups requiring special attention to include women who are poor, uneducated/illiterate, young, and living in rural areas. If this is how vulnerable women is being defining in this article, this should be clearly noted under the study population discussion.

**Question 2: Is the study design appropriate and is the work technically sound?**

 The design is a two-part descriptive analysis: one, based on an analysis of multiple datasets and two, literature review-based case studies of the 6 countries. The selection of factors for the first analysis assumes that the indicator mDFPS (modern contraceptive use prevalence – also called coverage) may be affected by changes over time in population levels of extreme poverty, gender inequality, and contraceptive method mix (short, long, permanent) and inequalities in household wealth, women’s age, and their level of education. The paper would benefit from the authors explaining in a little more detail why these were selected as factors affecting coverage (in part, referring to the need for a definition of vulnerable).

 In the background section, the authors note how lack of knowledge about family planning practices and access to contraceptive methods do not seem to be the main barriers to contraceptive use, even in the world’s poorest countries. Rather, they note that family planning is strongly dependent on beliefs and practices reflecting local social and cultural norms which vary widely across contexts. This is an insightful comment justifying more research/assessments into relevant social and cultural norms. Reference to the importance of social norms was a conclusion of their 2018 sister article. To this point, I was anticipating that the design and analysis of this paper would delve deeper into an assessment of specific social and cultural norms affecting modern family planning use coverage. Gender inequality, one of the contextual factors studied, reflects persistent/prevalent norms and it is understood that the authors were constrained by the availability of quantitative data in terms of their selection of factors to analyze. However, to link more closely to the background rationale for this study, I suggest that the authors expand their description of contextual factors to highlight how these factors qualify as social/cultural influences and where their study specifically addresses social and cultural norms. The paper would similarly benefit from such an explanation being incorporated more clearly into the introduction of the case study section. 

 Regarding the latter, the authors note that country case studies were included to provide additional political and sociocultural context. However, the two were written as disparate descriptive analyses without much cross reference. If each case study aims to help interpret the results of their initial analyses, I would consider reversing the flow of the paper so that the case studies come first. Either way, I suggest that there be some/more cross reference between the two write ups to help the reader better see connections between the two parts, thereby justifying the two-part design of the paper. 

**Question 3: Are sufficient details of methods and analysis provided to allow replication by others?**

 The authors provide good detail on the formula used for their coverage indicator (mDFPS) and define most terms well for the reader. One exception is the authors’ use of CMPO in their mDFPS estimate formula to represent modern contraceptive use prevalence. It would be useful for this definition to be noted more clearly in a formula footnote. Of note, to better understand some study decisions, I had to periodically refer to the sister article in which they also defined/clarified some of the methods used and results that informed this article.

 The authors provide a detailed list of which methods were considered modern in their analysis and a reference for this decision (#28).  Of note, this list does not concur with more recent definitions by WHO and the DHS viz inclusion of some methods e.g., fertility awareness methods such as Standard Days - a discrepancy noted by the authors. Given this discrepancy, and the potential for different levels of readership familiarity with the changing definition of modern methods over time, it would be useful for the authors to note *higher up* in the article (in the text or a footnote) that differences exist in which methods are now considered modern by different researchers and /or have been historically included in mCPR, as well as the authors’ rationale for excluding methods now currently considered modern by some researchers.

 It is assumed that the values incorporated in their mDFPS formula for CMPO and method-specific use were derived anew from the source datasets (not the aggregate and method-specific mCPR initially calculated by other researches using the same datasets). However, the paper would benefit from the authors clarifying this as values for modern contraceptive use prevalence will differ in different reference documents, reflecting differences in what is considered a modern method. Similarly, the indicator CPR initially analyzed from source datasets may have had a different denominator (married/women in union) versus all sexually active women. The authors note this difference, but the paper could benefit from this being clarified more within the methods section.

**Question 4: If applicable, is the statistical analysis and its interpretation appropriate?**

 This is a descriptive, ecological study and thus no tests for statistical associations were involved.

 However, in Part 1, statistical analyses were used to develop a wealth score, assigned to each household in which women were interviewed, divided into five, equally sized groups, weighted by the number of household residents. The wealth score involved principal component analyses, estimated separately according to area of residence (as they felt that relevant assets could vary by area), later combined into a single score using a regression-based scaling procedure. The authors noted that they considered the survey design in all these analyses (conducted in Stata 16.0) although it is not clear what is meant by this in the context of developing the wealth score. It would thus be useful if the authors expanded briefly on this aspect of their analysis. 

 The authors use scatter plots to explore changes over time in the indicator mDFPS along with changes in the proportion of the total population living in extreme poverty and population levels of gender inequality. The authors next use graphs and bar charts to evaluate changes over time in mDFPS against changes in the mix of contraceptive methods used in each country, as well as inequalities (according to household wealth, women’s age, and women’s education) derived from source datasets. The use of scatter plots and graphs/bar charts is a useful means of presenting the results of descriptive analyses. Also, the equiplot graphs allow readers to more easily grasp trends in inequalities and absolute inequality, as represented by the distance between subgroups analyzed.

 Interpretation of the figures was straightforward and appropriately represented what the figures revealed. 

**Question 5: Are all the source data underlying the results available to ensure full reproducibility?**

 The authors provide a full list of the original data sources and where/how they obtained access.

**Question 6: Are the conclusions drawn adequately supported by the results?**

 In places, the authors say that there was concomitant increase in coverage and select contextual factors which is appropriate wording.  In other places, the authors say a factor *contributed* to coverage increase which is not fully borne out by the methods employed. More appropriate wording would be to say that the factors potentially or possibly contributed to the observed changes in coverage.

Is the work clearly and accurately presented and does it cite the current literature?

Partly

If applicable, is the statistical analysis and its interpretation appropriate?

Yes

Are all the source data underlying the results available to ensure full reproducibility?

Yes

Is the study design appropriate and is the work technically sound?

Partly

Are the conclusions drawn adequately supported by the results?

Partly

Are sufficient details of methods and analysis provided to allow replication by others?

Yes

Reviewer Expertise:

Public health practitioner and epidemiologist with expertise in factors affecting family planning programming, barriers and use in low-resource settings globally.

I confirm that I have read this submission and believe that I have an appropriate level of expertise to confirm that it is of an acceptable scientific standard, however I have significant reservations, as outlined above.

## References

[1] : Demand for family planning satisfied with modern methods among sexually active women in low- and middle-income countries: who is lagging behind?. *Reprod Health* .2018;15(1) : 10.1186/s12978-018-0483-x 42 10.1186/s12978-018-0483-x 29510682PMC5840731

# Peer review: Reviewer response for version 2

**DOI:** 10.21956/gatesopenres.15400.r32680

**Published:** 2022-10-17

**Authors:** Carmen Simone Grilo Diniz

**Affiliations:** 1 Department of Health, Life Cycles and Society, Public Health School, University of São Paulo, São Paulo, Brazil

The revised text is in line with the suggestions made. I am satisfied with the end result.

Is the work clearly and accurately presented and does it cite the current literature?

Yes

If applicable, is the statistical analysis and its interpretation appropriate?

I cannot comment. A qualified statistician is required.

Are all the source data underlying the results available to ensure full reproducibility?

Yes

Is the study design appropriate and is the work technically sound?

Yes

Are the conclusions drawn adequately supported by the results?

Yes

Are sufficient details of methods and analysis provided to allow replication by others?

Yes

Reviewer Expertise:

Public Health, Reproductive Health and Rights, Gender, Maternal health

I confirm that I have read this submission and believe that I have an appropriate level of expertise to confirm that it is of an acceptable scientific standard.

# Peer review: Reviewer response for version 1

**DOI:** 10.21956/gatesopenres.14839.r32468

**Published:** 2022-10-10

**Authors:** Carmen Simone Grilo Diniz

**Affiliations:** 1 Department of Health, Life Cycles and Society, Public Health School, University of São Paulo, São Paulo, Brazil

The article “Learning from success cases: ecological analysis of pathways to universal access to reproductive health care in low- and middle-income countries” begins by considering that access to family planning is a human right and that different countries and national international organizations have committed to expanding its coverage. The objective of the article was to investigate which contexts facilitate the implementation of these policies in recent decades.

 The authors analyzed data from surveys conducted in Egypt, Ethiopia, Rwanda, Afghanistan, Brazil and Ecuador, based on publicly available data. They analyze the characteristics of different contexts in terms of women's empowerment, degrees of extreme poverty, and types of contraceptives. Among the findings, they show that the use of modern methods of contraception varies from 38% in Afghanistan to 94% in Brazil, and is related to both gender inequality and extreme poverty.

 They conclude that increased use of modern methods of contraception was associated with reduced poverty and gender inequality.

 My only suggestion is a change in the title: instead of “Learning from success cases: ecological analysis of pathways to universal access to reproductive health care in low- and middle-income countries” it should be “Learning from success cases: ecological analysis of pathways to universal access to family planning care in low- and middle-income countries”, as this is the only aspect of the vast topic of reproductive health they address.

Is the work clearly and accurately presented and does it cite the current literature?

Yes

If applicable, is the statistical analysis and its interpretation appropriate?

I cannot comment. A qualified statistician is required.

Are all the source data underlying the results available to ensure full reproducibility?

Yes

Is the study design appropriate and is the work technically sound?

Yes

Are the conclusions drawn adequately supported by the results?

Yes

Are sufficient details of methods and analysis provided to allow replication by others?

Yes

Reviewer Expertise:

Public Health, Reproductive Health and Rights, Gender, Maternal health

I confirm that I have read this submission and believe that I have an appropriate level of expertise to confirm that it is of an acceptable scientific standard, however I have significant reservations, as outlined above.
